# Application of the uridine auxotrophic host and synthetic nucleosides for a rapid selection of hydrolases from metagenomic libraries

**DOI:** 10.1111/1751-7915.13316

**Published:** 2018-10-09

**Authors:** Nina Urbelienė, Simonas Kutanovas, Rita Meškienė, Renata Gasparavičiūtė, Daiva Tauraitė, Martyna Koplūnaitė, Rolandas Meškys

**Affiliations:** ^1^ Department of Molecular Microbiology and Biotechnology Institute of Biochemistry Life Sciences Center Vilnius University Sauletekio 7 Vilnius LT‐10257 Lithuania

## Abstract

A high‐throughput method (≥ 10^6^ of clones can be analysed on a single agar plate) for the selection of ester‐hydrolysing enzymes was developed based on the uridine auxotrophy of *Escherichia coli* strain DH10B *ΔpyrFEC* and the acylated derivatives 2′,3′,5′‐*O*‐tri‐acetyluridine and 2′,3′,5′‐*O*‐tri‐hexanoyluridine as the sole source of uridine. The proposed approach permits the selection of hydrolases belonging to different families and active towards different substrates. Moreover, the ester group of the substrate used for the selection, at least partly, determined the specificity of the selected enzymes.

## Introduction

Discovery of novel enzymes and their engineering is the basis of the tremendously fast‐developing industrial biotechnology. Enzyme‐based organic synthesis constantly needs innovative, diverse and robust biocatalysts (Bornscheuer, 2018). Ester hydrolases (EC 3.1), commonly known as esterases or lipases, represent a diverse group of hydrolases catalysing the cleavage and formation of ester bonds (Jaeger and Eggert, 2002; Dimitriou *et al*., 2017; Bornscheuer, 2002). Hydrolases have a growing number of applications in biotechnology, particularly in fine chemicals industries, because of their enantioselectivity and regioselectivity. Catalytic promiscuity, no requirement for cofactors as well as stability and activity in organic solvents also stimulate interest in the aforementioned enzymes (Jaeger and Eggert, 2002; Bornscheuer and Kazlauskas, 2004; Rauwerdink and Kazlauskas, 2015; Martínez‐Martínez *et al*., 2018).

The hunt for novel enzymes, including esterases, is usually based on one of the following strategies: (i) construction of metagenomic libraries, and subsequent functional screening (Martínez‐Martínez *et al*., 2018; Ferrer *et al*., 2015; Taupp *et al*., 2011; Peña‐García *et al*., 2016; Ferrer *et al*., 2016; Steele *et al*., 2009), (ii) (meta)genome mining based on the homology analysis, chemical synthesis of the target genes and evaluation of the catalytic properties of the recombinant proteins (Martínez‐Martínez *et al*., 2018), (iii) directed evolution, including a high‐throughput scale (HTS) microfluidics screening of the combinatorial libraries of randomly generated enzyme variants (Colin *et al*., 2015; Bunzel *et al*., 2018), (iv) rational *in silico* design followed by an experimental verification of selected variants (Santiago *et al*., 2018; Packer and Liu, 2015; Bornscheuer *et al*., 1999; Fernández‐Álvaro *et al*., 2011).

Novel powerful techniques notwithstanding, the functional metagenome screening remains an interesting and promising option, since it offers a possibility of discovering enzymes with unique properties and novel scaffolds applicable for further evolution *in vitro*. However, the number of tested clones and positive hits strongly depends on the screening method applied, as well as on the substrate used and can vary from several thousands to billions with a success rate ranging from 1:11 to 1:193 200, in the case of ester hydrolases (Peña‐García *et al*., 2016; Ferrer *et al*., 2016).

At present, a functional screening of esterases is usually performed either by employing chromogenic substances (e.g. *p*‐nitrophenyl esters) or by using tributyrin‐supplemented agar plates (Martínez‐Martínez *et al*., 2018; Peña‐García *et al*., 2016). In addition, other esters, various fluorogenic substrates or enzyme cascades have been applied for the identification of ester hydrolases‐producing microorganisms (Peña‐García *et al*., 2016; Rossum *et al*., 2013). Although both low‐throughput and robotic‐based HTS systems are being developed, the exploitation of the genotype–phenotype linkage or of the genetic selection is not very common (Reetz *et al*., 2008). Mutations in the genes of host cell metabolism can be used for discovery of specific enzymes (Forney *et al*., 1989), their analysis (Delauney *et al*., 1993) and for the design of antibiotics‐free (Dong *et al*., 2010) or catalytic antibodies‐based selection systems (Smiley and Benkovic, 1994). Since only a few of such systems have been developed for the selection of ester hydrolases, the hydrolysis reaction, which liberates glycerol as a carbon source, has been applied for the generation of active esterase mutants (Bornscheuer *et al*., 1998). The appropriate aspartate esters and the *Escherichia coli* strain, in which both pathways leading to the synthesis of aspartate are blocked, have been used to select mutants of *Bacillus subtilis* lipase A (Boersma *et al*., 2008). Also, a mixture of isosteric (*R*)‐ and (*S*)‐enantiomers has been used as a substrate in the bond‐breaking reaction that generates a growth‐promoting energy source and a growth‐inhibiting compound (Reetz and Rüggeberg, 2002). However, a wider application of such system is slightly restricted, since the starting substrates must be non‐toxic to the host organism. In the case of esterase‐based generation of energy and/or carbon source, high concentrations of the substrates have to be applied to support a satisfactory growth of the positive clones (Acevedo‐Rocha *et al*., 2014).

Recently, it has been shown that the uracil auxotrophic strain of *E. coli* can be successfully used for an identification of the specific genes encoding the catabolism of the modified uracil base(Aučynaitė *et al*., 2018). Here, we present a method of functional screening of hydrolases based on uridine auxothrophy and the appropriate uridine derivatives that serve as substrates for the ester‐hydrolysing enzymes. The proposed method combines the best features of the genotype–phenotype linkage and the flexibility in the chosen substrate hence allowing an efficient selection of esterases from metagenomics libraries. Compared with the known methods developed for screening of esterases/lipases, the proposed selection system has many advantages including a rapidness, a high throughput (millions of clones can be analysed by using a single agar plate) and possibility to apply different substrates for identification of the enzymes with the desired catalytic properties.

## Results and discussion

**Figure 1:**
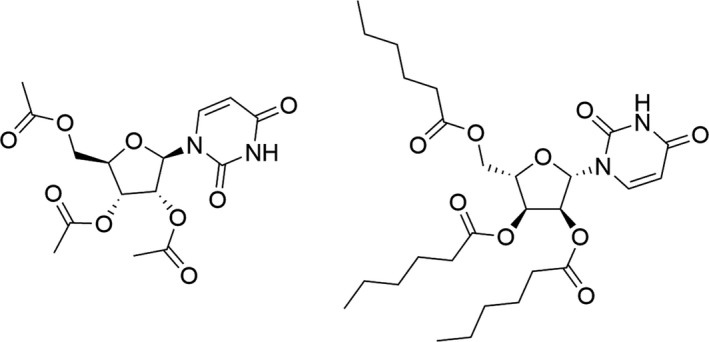
2′,3′,5′‐tri‐O‐acetyluridine (**1**) or 2′,3′,5′‐tri‐O‐hexanoyluridine (**2**).

**Figure 2:**
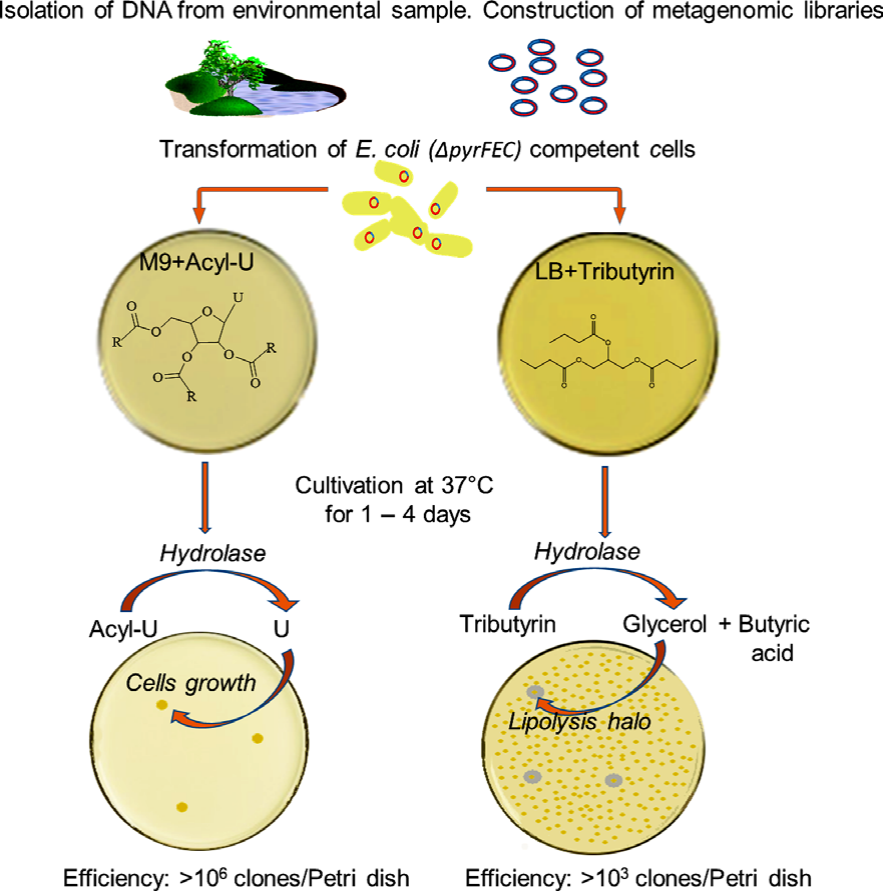
The principle for the selection of ester‐hydrolysing enzymes. Metagenomic DNA isolated from the environmental samples is fragmented, inserted into an appropriate vector and used to transform the competent cells of *E. coli* DH10B Δ*pyrFEC*::Km. A mineral medium containing 2′,3′,5′‐tri‐*O*‐acetyluridine or 2′,3′,5′‐tri‐*O*‐hexanoyluridine as the sole source of uridine is used to select the clones exhibiting acylesterase activity (left column). The positive hits complement the uridine auxotrophy of the *E. coli* DH10B *ΔpyrFEC::*Km strain by hydrolysis of the substrate (compound **1** or compound **2)** to uridine allowing the colony formation. A standard tributyrin agar plate method is represented on the right.

To develop the selection method, 2′,3′,5′‐tri‐*O*‐acetyluridine – and 2′,3′,5′‐tri‐*O*‐hexanoyluridine (Fig. 1) – hereafter indicated respectively as compound **1** or **2** throughout the text were chosen as the sole source of uridine, supporting growth only of those recombinant clones (Fig. S1), which encode ester hydrolases that complement the uridine auxotrophy of the *E. coli* DH10B Δ*pyrFEC*::Km (Aučynaitė *et al*., 2018) strain by hydrolysis of compound **1** or **2** to uridine (Fig. S1). Due to location of the *pyr* genes in different parts of the chromosomes of many bacteria, the rationale of using *E. coli* mutant harbouring triple mutations in the genes encoding the pathway of uridine biosynthesis was to lower a level of the false‐positive hits. Regarding the application of two structurally different substrates used for the selection, it was supposed that the uridine derivatives with a varied acyl length could predetermine the properties of the selected hydrolases, and the enzymes with a different preference to acyl size would be identified. The principle of the selection method is shown in Fig. 2.

The metagenomic DNA isolated from different soil samples was partially digested with several restriction endonucleases, and the fragmented DNA was used for the construction of metagenomics libraries. In total, 19 libraries (Table S1) were tested. Clones exhibiting acylesterase activity were selected on MD medium containing 100 μg ml^−1^ ampicillin and 40 μg ml^−1^ kanamycin, as well as 20 μg ml^−1^ of the compound **1** or **2** (Fig. 1). In total, 87 positive clones were selected. All clones were re‐streaked on MD medium without uridine or uridine derivative. Four of the selected hits capable of forming colonies on MD medium without the compound **1** or **2** were considered as false positives (approximately 5%) and were omitted from the further analysis. The plasmid DNA from the remaining clones was isolated, and the fragments obtained after restriction digestion were analysed by sequencing to exclude the redundancy. Hence, 30 clones were chosen for further analysis.

Bioinformatics analysis showed that the selected clones exhibiting esterase activity contained ORFs with medium (31%) to high (100%) sequence identity to proteins found in the databases (Table 1). Ten of the closest homologs were annotated as hypothetical proteins.

**Table 1:** The list of selected ester hydrolases

Clone, GenBank accession No.	The nearest homolog, GenBank accession No.	Identities (%)
24T5, MH423251	*Kaistia soli*, WP_073056985.1, alpha/beta hydrolase	67
24T1, MH423255	*Devosia epidermidihirudinis*, WP_046138431.1, hypothetical protein WH87_03740	99
24T3, MH423254	*Devosia riboflavin*, WP_035086921.1, hypothetical protein	95
33T1, MH423252	*Devosia* sp. Root685, WP_082561207.1, hypothetical protein	95
30T1, MH423263	*Sinorhizobium* sp. GL2, KSV78272.1, hypothetical protein N182_21555	61
30T2, MH423266	*Acidobacteria bacterium*, OLB17 KXK06970.1, Alpha/beta hydrolase	74
36T2, MH423275	*Paracoccus aminophilus*, WP_020950583.1, alpha/beta hydrolase	74
BD1, MH423279	*Rhizobium tropici*, WP_015341790.1, alpha/beta hydrolase fold‐3 catalytic domain‐ containing protein	51
EN3H*, MH423261	*Pseudomonas lini*, WP_048393178.1, alpha/beta hydrolase	95
MO101T, MH423257	*Lysinibacillus* sp. AR18‐8, WP_066036519.1, alpha/beta hydrolase	99
CAP3H*, MH423259	MULTISPECIES: Cupriavidus, WP_092295063.1, alpha/beta hydrolase	96
BD2H*, MH423277	MULTISPECIES: Duganella, WP_090189706.1, alpha/beta hydrolase	70
3T, MH423256	*Roseiflexus* sp. RS‐1, WP_011955564.1, acetylxylan esterase	61
GRU1, MH423265	*Paenibacillus phocaensis*, WP_068787184.1, acetylesterase	100
SVG3, MH423258	*Rheinheimera* sp. KL1, WP_053423444.1, alpha/beta hydrolase	93
K3H2*, MH423271	*Dehalococcoidia bacterium*, PWB48329.1, hypothetical protein C3F10_01030	67
4H1T, MH423260	*Sphingobacterium mizutaii*, WP_093095847.1, esterase family protein	87
RIEB, MH423273	*Rhizobium* sp. P44RR‐XXIV, WP_077472732.1, alpha/beta hydrolase	68
SVGPA2T, MH423270	*Sphingopyxis* sp. C‐1, WP_062186324.1, alpha/beta hydrolase	93
C233, MH423278	*Paracoccus* sp. TRP, WP_010397925.1, glycoside hydrolase	74
SVG1, MH423269	*Ensifer* sp. LC163, WP_083222508.1, serine hydrolase (Beta‐lactamase)	92
12T, MH423253	*Acidobacteria bacterium* RIFCSPLOWO2_12_FULL_67_14b, OFW37874.1, hypothetical protein A3J29_14090	65
45T3, MH423262	*Brevundimonas* sp. Leaf363, WP_056098529.1, ribosomal‐protein‐alanine N‐acetyltransferase	67
PLA1, MH392251	*Gemmata* sp. SH‐PL17, AMV27246.1, GDSL‐like Lipase/Acylhydrolase	58
33T3, MH423272	*Pseudohongiella acticola*, WP_047492018.1, hypothetical protein	61
BD9, MH423268	*Mesorhizobium temperatum*, WP_095491896.1, SGNH/GDSL hydrolase family protein	50
36T1, MH423267	*Firmicutes bacterium* CAG:272, CDC74944.1, sialate O‐acetylesterase	31
MO4B, MH423274	*Microvirga ossetica*, WP_099513428.1, peptidase	92
EN1H*, MH423276	*Bacillus* sp. J33, WP_026581439.1, hypothetical protein	100
1315H*, MH423264	*Microbacterium gorillae*, WP_094770426.1, DUF998 domain‐containing protein	57

The clones selected using compound **2** as the uridine source are marked by asterisk.

**Figure 3:**
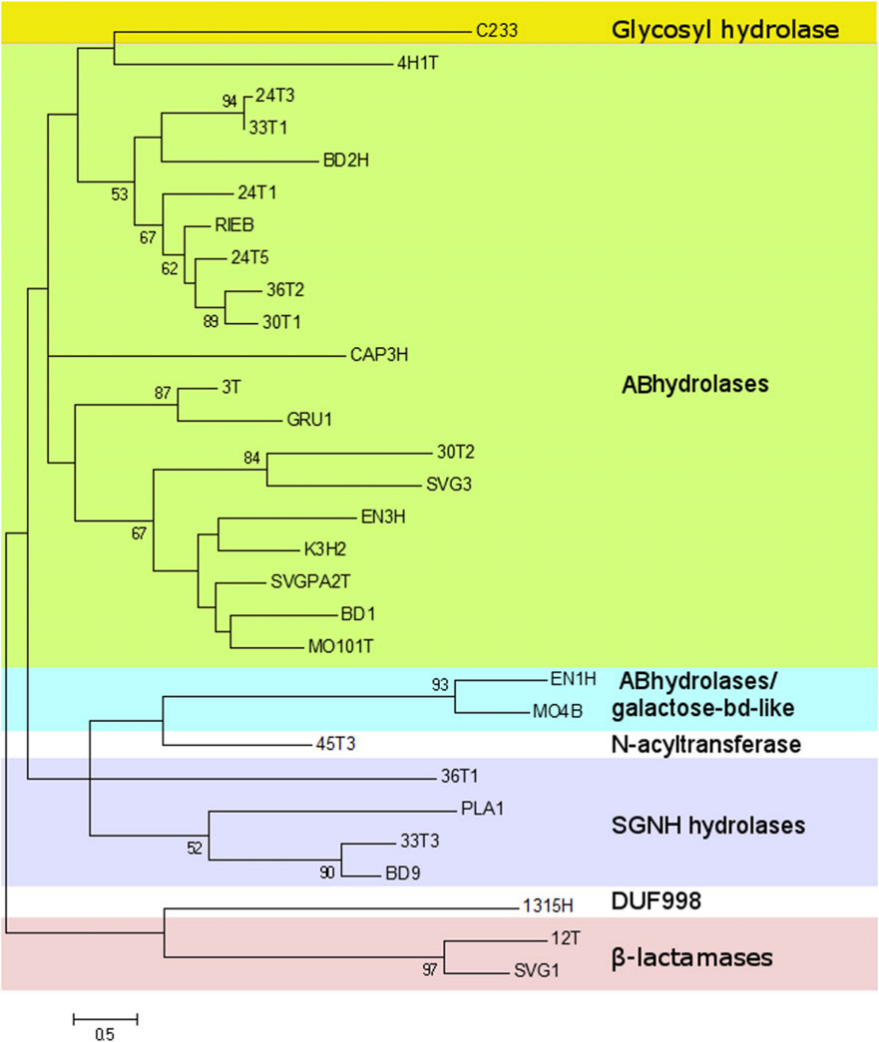
Phylogenetic tree of the selected esterases. The phylogenetic analysis of metagenome fragments was conducted using the *Maximum Likelihood Tree* routine of mega 7 software. The alignment was performed using clustalw. The tree with the highest log likelihood (−7599.8830) is shown. Only bootstrap values higher than 50% are indicated.

The phylogenetic analysis of the selected hydrolases showed that the enzymes represent very diverse groups of proteins (Fig. 3). Most of the hits were representatives of ABhydrolase (α/β hydrolase) superfamily (SSF53474) (19 hits) and SGNH hydrolases (SSF52266) (4 hits) followed with proteins belonging to β‐lactamases (SSF56601) (2 hits), α/β hydrolases/galactose‐binding domain‐like (2 hits), glycosyl hydrolases (SSF51445) (1 hit), *N*‐acyltransferase superfamily (SSF55729) (1 hit) and one DUF998 family protein (Fig. 3). All identified proteins of the ABhydrolases group had a conserved Gly‐x‐Ser‐x‐Gly catalytic motif (Bornscheuer, 2002) and were distributed among different families: α/β hydrolase‐1, α/β hydrolase‐3 and peptidase S9 (Fig. S2A). Two esterases MO4B and EN1H consisted of two domains – ABhydrolase and galactose‐binding‐like domain. The common function of these domains is to bind to specific ligands, such as cell‐surface‐attached carbohydrate substrates. Both hydrolases had the conserved motif of serine proteases (Delauney *et al*., 1993), and the consensus sequence surrounding the active‐site serine had been identified as G‐X‐S‐Y‐X‐G (Fig S2B). Based on the phylogenetic and BLAST analyses, the hits 33T3, BD9, PLA1 and 36T1 belonged to SGNH hydrolase superfamily. The catalytic Ser‐His‐Asp (Glu) triad (Polgár, 2005) was determined in the amino acid sequences of esterases 33T3, BD9, PLA1, but no such motif was found in 36T1 (Fig. S2C). Two esterases 12T and SVG1 were similar to β‐lactamases and had the conserved S‐X‐X‐S (Wagner *et al*., 2009) and LLXHXXG motifs (Ranjan *et al*., 2005) of Esterase VIII, but two other highly conserved β‐lactamase motifs (Y‐A‐N) and (K‐T/S‐G) (Joris *et al*., 1988) were not found (Fig. S2D). Signal peptide sequence analysis showed the presence of signal peptides in 17 out of 30 selected esterases (Table S3). Various ABhydrolases, SGNH hydrolases and β‐lactamases were previously isolated from the metagenomic libraries using tributyrin or other method of functional screening(Popovic *et al*., 2017). However, in addition to the novel variants of known groups of hydrolases, the method presented here also allowed the selection of novel scaffolds, such as C233, 45T3 and 1315H. The protein C233 belonged to PF00933 (Glyco_hydro_3) family of glycoside hydrolases (Bourne and Henrissat, 2001) with unknown esterolytic activity. The hydrolase 45T3 belonged to the family PF00583 and was similar to a ribosomal‐protein‐alanine *N*‐acetyltransferase (Yoshikawa *et al*., 1987) Hu *et al*., 2010; Jones and O'Connor, 2011). The most interesting hit 1315H encoded a protein with a DUF998 domain. The protein sequence analysis using SMART (http://smart.embl-heidelberg.de/) revealed that the enzyme 1315H may be a transmembrane protein without any predictable function.

Thus, a functional selection of metagenomic libraries revealed enzymes from very diverse protein families, including several hits that, based on bioinformatics, could not be annotated as hydrolases active towards esters.

To confirm that the hits encoded the enzymes with esterolytic activity, the selected genes were PCR‐amplified, and the resulting fragments were ligated into pET21a or pLATE31 expression vectors. *E. coli* strain BL21 (DE3) was transformed with the recombinant plasmids and used for the expression of the recombinant proteins. In total, 27 recombinant proteins were purified by Ni‐NTA chromatography (Fig. S3), and 23 of them showed purity higher than 90% (Table 2). Due to hydrophobic nature, the protein encoded by the clone 1315H was not purified, and an insoluble fraction of the cells was used for the determination of activity. The proteins encoded by clones RIEB and 4H1T were not purified due to a poor expression. The purity of the protein MO4B was not analysed because the concentration of the purified enzyme was very low due its poor solubility. During overexpression, sufficient amounts of five proteins 24T1, 24T3, 33T1, SVG1 and SVG3 were released into the extracellular space, and the enzymes were purified from the medium, most likely, without the signal peptides.

**Table 2:** The activity of the recombinant esterases towards *p*‐ nitrophenyl esters

Clone	Purity, %	Activity, U μg^−1^ of protein			
		pNP‐acetate	pNP‐butyrate	pNP‐valerate	pNP‐decanoate
24T5	96	38 ± 3.8	26.4 ± 4.8	38.2 ± 3.8	0.3 ± 0.01
24T1	94	66.6 ± 10.7	10 ± 20.8	84 ± 8.7	0.9 ± 0.01
24T3	85	45 ± 11	46 ± 8.1	60.5 ± 6.2	–
33T1	96	49 ± 0.8	80 ± 2.4	27.5 ± 6.5	0.9 ± 0.01
30T1	93	55 ± 2.3	54.4 ± 5.8	20 ± 2.1	–
30T2	93	52 ± 4.7	8 ± 0.1	2.6 ± 0.8	–
36T2	77	32.7 ± 2.9	4.8 ± 0.3	2.2 ± 0.25	–
BD1	93	12.9 ± 0.3	5.7 ± 0.8	4.3 ± 0.5	–
EN3H	93	283 ± 15	336 ± 15	196 ± 18	8.6 ± 0.6
MO101T	96	159 ± 19	0.2 ± 0.1	–	–
CAP3H	74	29.1 ± 6.2	75 ± 10	34.3 ± 6.5	10.3 ± 0.34
BD2H	93	1.9 ± 0.6	18 ± 0.5	24.3 ± 1.1	3.1 ± 0.5
3T	93	23 ± 4	0.9 ± 0.2	–	–
Gru1	98	177 ± 0.3	2.7 ± 0.8	1.3 ± 0.5	–
SVG3	99	346 ± 7.5	35 ± 9.6	33.6 ± 3.6	–
K3H2	98	349 ± 35	425 ± 21	49.3 ± 2.5	16.9 ± 2.7
4H1T^a^	n.a.	n.a.	n.a.	n.a.	n.a.
RIEB^a^	n.a.	n.a.	n.a.	n.a.	n.a.
SVGPA2T^a^	n.a.	n.a.	n.a.	n.a.	n.a.
Tb107T^b^	94	122 ± 4.7	133 ± 8.8	7.4 ± 0.6	2.6 ± 1.4
C233	90	3.7 ± 0.5	5.8 ± 1.4	4.7 ± 1.6	–
SVG1	95	343 ± 15	367 ± 15.8	189 ± 52.8	2.1 ± 0.34
12T	77	4.1 ± 0.5	0.3 ± 0.2	0.3 ± 0.2	–
45T3	86	104 ± 12	78 ± .5.4	49.5 ± 6.1	–
PLA1	93	30 ± 0.8	2.4 ± 0.02	–	–
33T3	82	80 ± 6.7	7.6 ± 0.7	0.9 ± 0.4	–
BD9	69	3.2 ± 1.4	3 ± 2	3.2 ± 2.6	–
36T1	95	4.5 ± 0.5	6 ± 0.7	6.7 ± 3	–
MO4B^c^	–	1.3 ± 0.1	0.12 ± 0.1	–	–
EN1H	95	14.4 ± 2	47 ± 17	74.4 ± 8.9	2.0 ± 0.2
1315H^d^	–	2.2 ± 1.3	17.1 ± 10	35.6 ± 17.4	1.±0.6

n.a., not analysed; –, no activity.

a The proteins were not purified due to a poor expression.

b Tb10_7T was screened on the tributyrin agar plate.

c A concentration of the protein was too low for a purity evaluation.

d The protein was expressed but not purified. The activity calculated based on proteins of crude extract.

The hydrolytic activity of the purified proteins was analysed with various *p*‐nitrophenyl (pNP) esters: acetate, butyrate, valerate, decanoate, palmitate and stearate. All hydrolases were active towards the short‐chain esters pNP‐acetate and pNP‐butyrate, most of them used pNP‐valerate, but only a few of them hydrolyzed pNP‐decanoate (Table 2). Neither pNP – palmitate or stearate was recognized as the substrate (data not show).

The esterase C233 exhibited activity towards short‐chain pNP esters but did not hydrolyze the established substrates of glycoside hydrolases, such as 4‐nitrophenyl derivatives of α‐l‐arabinofuranoside, α‐ and β‐l‐arabinopyranoside, α‐ and β‐d‐xylopyranoside or β‐d‐glucopyranoside.

The recombinant enzymes selected using compoud **1** were able to hydrolyze short‐chain esters of pNP with a high efficiency, but displayed a very weak activity against pNP‐decanoate. Only five enzymes from the 22 selected hydrolyzed pNP‐decanoate, although three of them (24T5, 21T1 and 33T1) exhibited a very weak activity against this ester. Six enzymes MO101T, SVG3, GRU1, PLA1, 3T and 33T3 exhibited a strong preference for pNP‐acetate as the substrate (Table 2).

The enzymes selected on compound **2** demonstrated the activity towards the longer‐chain esters. These esterases hydrolyzed pNP‐decanoate with a high efficiency (Table 2), however the highest specific activity in this group of hydrolases was towards pNP‐butyrate (EN3H, K3H2, CAP3H) or pNP‐valerate (EN1H, 1315H) (Table 2). These results indicated that the bulkiness of the ester group of the substrate used for the selection, at least partly, determined the specificity of the selected enzymes. These findings may be useful for the development of a more effective direct selection of the enzymes with the desired properties.

**Figure 4:**
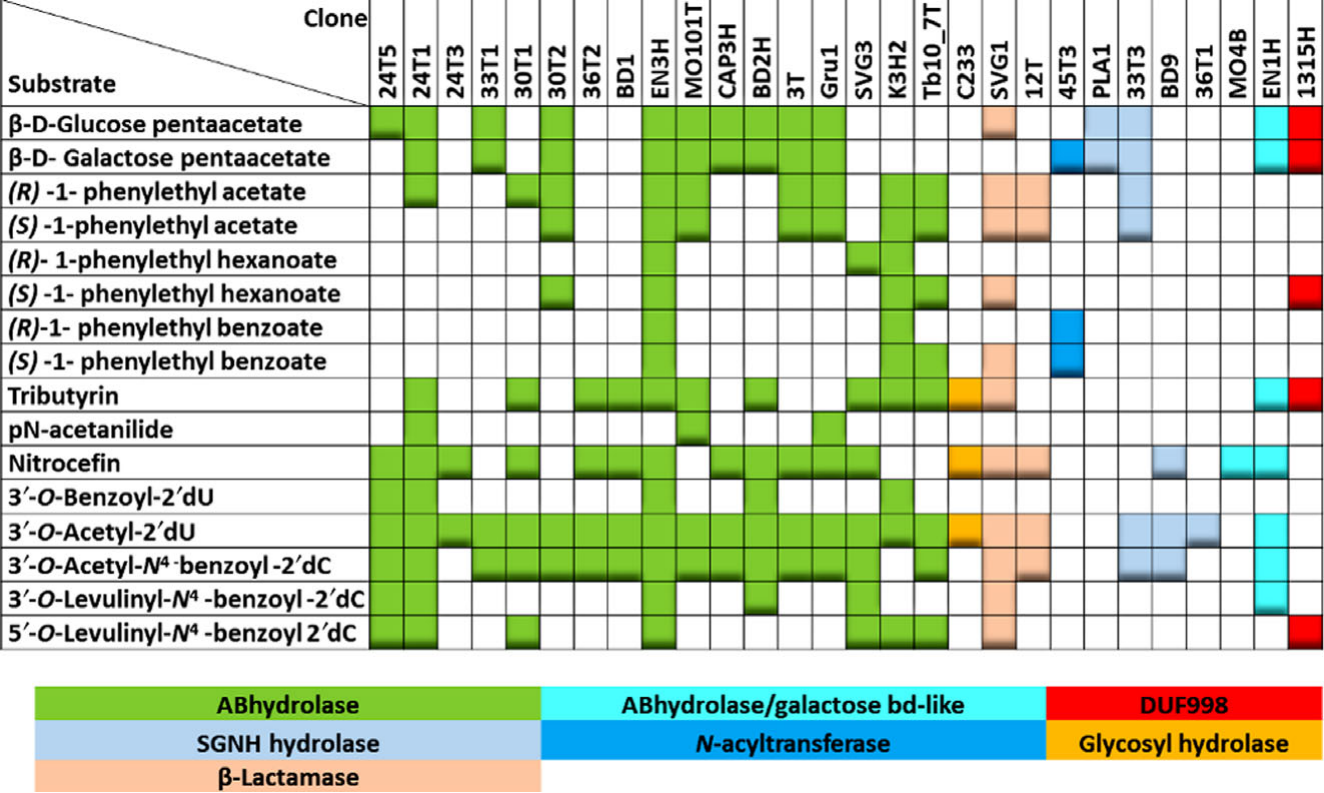
The substrate specificity of the recombinant esterases.

Further analysis of the substrate specificity of the selected enzymes showed that approximately half of the tested esterases could accept the bulky peracetylated carbohydrates, and thirteen esterases could hydrolyze tributyrin (Fig. 4) that was confirmed by the cultivation of those clones on tributyrin agar (Fig. S4). Such results definitely showed that only the fraction of hits would be screened using a standard tributyrin agar approach.

To test if the standard selection on agar plates with tributyrin would result in clones exhibiting activity towards compoud **1** or compound **2**, several metagenomics libraries were assessed, and two hits (Tb7_1T and Tb10_7T) forming the halos indicative of the hydrolysis were screened. Tb7_1T (MH423281) encoded the group β‐lactamases protein, and Tb10_7T (MH423280) was most similar to ABhydrolases. Neither of the two clones was capable of growing on compound **1** or **2** as a uridine source. Tb7‐1T was not further analysed because of its insolubility. The purified protein Tb10_7 was not only active towards pNP esters (Table 2), but also hydrolyzed 3′‐*O*‐acetylated nucleosides *in vitro* (Fig. 4). Moreover, different activity profiles of identified esterases towards monoacylated nucleosides harbouring short, aromatic and bulky aliphatic groups (Fig. 4) suggest that a more selective system may be designed and applied, for example, for a substrate‐guided isolation and tailoring of the desired mutants. To determine enantioselectivity of the esterases, *R*/*S*‐1‐phenylethyl esters with varied length of the ester groups were synthesized and applied for analysis (Fig. 4). The enzymes 24T1 and 30T1 were enantioselective towards *R*‐1‐phenylethyl acetate. Four (30T2, SVG1, 1315H, Tb10‐7T) and two (SVG1, Tb10_7T) esterases preferred *S*‐1‐phenylethyl hexanoate and *S*‐1‐phenylethyl benzoate respectively. No esterase was enantioselective towards *R*‐1‐phenylethyl hexanoate or benzoate. Furthermore, to analyse the promiscuity of the selected esterases, several amides were tested as substrates. Consequently, three esterases were found to hydrolyze acetanilide, and 18 were active towards nitrocefin (Fig. 4).

In conclusion, a combination of uridine esters (compound **1** and **2**) and the uridine auxotroph mutant Δ*pyrFEC* of *E. coli* allows for a functional selection of novel ester hydrolases from metagenomic libraries. Evidently, the selection method presented here is highly complementary to traditional approaches and is applicable for allowing the discovery of novel esterases with different structural and catalytic characteristics. Compared with the known methods, the proposed selection system has many advantages: (i) it is a HTS method that allows for a rapid (1–4 days) processing of large (meta)genome libraries (≥ 10^6^ of clones can be analysed on a single agar plate) with low number of false positives, ii) depending on the selection media used, it permits the functional selection of hydrolases belonging to different protein families, (iii) it is flexible since, by using different substrates, for example, esters of the fatty acids of different lengths or bulky carboxylic acids, it allows the identification of enzymes with desired properties, (iv) it would be suitable for the selection of regioselective esterases if the appropriate substrate was used, (v) it allows the identification of novel proteins that previously have not been known as the ester‐hydrolysing enzymes or cannot be screened using, for example, a tributyrin method, (vi) the appropriate uridine auxotrophs are available for a wide spectra of microbial hosts, including extremophiles and yeasts or can be easily constructed, for example, using 5‐fluororotic acid selection (Boeke *et al*., 1987; Kondo *et al*., 1991) and/or recombineering (Xu *et al*., 2015; Aparicio *et al*., 2018) hence offering a possibility of a functional screening of enzymes under distinct conditions. Definitely, the desired host cannot be capable to hydrolyze the chosen uridine ester. Further applications and improvements of the presented selection system are in progress.

## Experimental procedures

### Materials

Restriction endonucleases, HindIII, BamHI, PstI, Phusion DNA polymerase, aLICator™ LIC Cloning and Expression System Kit 3, PageRuler^TM^ Prestained Protein Ladder were purchased from Thermo Fisher Scientific, Vilnius, Lithuania, Pierce™ Coomassie Plus (Bradford, UK) Assay Reagent and HisPur™ Ni‐NTA spin column were purchased from Thermo Fisher Scientific, Rockford, IL. pET21a Vector was purchased from Novagen (Madison, USA). Nutrient medium were purchased Roth, Germany. pNP‐acyl esters, tributyrin, β‐d‐glucose pentaacetate, β‐d‐galactose pentaacetate, and uridine were purchased from Sigma‐Aldrich. ‘ZR Soil Microbe DNA MidiPrep™’ was purchased from Zymo Research, Freiburg, Germany. Nitrocefin was purchased from Oxoid, UK. 3′‐O‐benzoyl‐2′‐deoxyuridine were purchased from Carbosynth, UK. 3′‐*O*‐acetyl‐2′‐deoxyuridine, 3′‐*O*‐acetyl‐*N* ^4^‐benzoyl‐2′‐deoxycytidine, 3′‐*O*‐levulinyl‐*N* ^4^‐benzoyl‐2′‐deocytidine, and 5′‐*O*‐levulinyl‐*N* ^4^‐benzoyl‐2′‐deocytidine were purchased from Jena Bioscience, Jena, Germany.

### Synthesis of 2′,3′,5′‐tri‐O‐acetyluridine (**1**) and 2′,3′,5′‐tri‐O‐hexanoyluridine (**2**)

2′,3′,5′‐*O*‐acetyluridine and 2′,3′,5′‐hexanoyluridine were prepared by adapting and modifying methods reported in the literature(García *et al*., 2002; Michihata *et al*., 2013; Gaubert *et al*., 2006).

#### Synthesis of 2′,3′,5′‐*O*‐acetyluridine (**1**)

A mixture of acetic anhydride (1.74 ml, 18.45 mmol) and sodium acetate (0.17 g, 2.05 mmol) was heated for 20 min at 90°C. Then uridine (1 g, 4.1 mmol) was added to the hot solution and stirred for 1 h. After the reaction was completed (TLC), the mixture was quenched with sodium bicarbonate and extracted with chloroform. The organic phase was dried (Na_2_SO_4_) and evaporated under reduced pressure. The residue was purified by column chromatography (silica gel, chloroform/methanol mixture, 10:0→10:1) to afford 2′,3′,5′‐*O*‐triacetyluridine (1.45 g (95%) as a white solid. UV (CH_3_OH) λ_max_ 257 nm. MS (ESI^+^): *m/z* 371.10 [M+H]^+^, 369.10 [M−H]^−^. ^1^H‐NMR (DMSO‐*d*_*6*_): δ = 2.11 (s, 3H, CH_3_), 2.13 (s, 3H, CH_3_), 2.15 (s, 3H, CH_3_), 4.36 (m, 3H, CH, CH_2_), 5.35 (m, 2H, CH), 5.81 (d, 1H, *J *=* *8.1 Hz, CH=CH), 6.05 (d, 1H, *J *=* *4.7 Hz, CH), 7.41 (d, 1H, *J *=* *8.2 Hz, CH=CH), 9.69 (s, 1H, NH). ^13^C‐NMR (DMSO‐*d*_*6*_): δ = 20.41, 20.51, 20.77, 63.18, 70.20, 72.72, 79.92, 87.46, 103.44, 139.36, 150.35, 163.00, 168.75, 169.67, 170.18. The NMR spectra were consistent with reported previously (Gaubert *et al*., 2006).

#### Mixture of mono‐, di‐ and tri‐*O*‐hexanoyluridine (**2**) synthesis

To a stirred mixture of uridine (0.24 g, 1 mmol) and triethylamine (0.7 ml, 5 mmol) in 1,4‐dioxane (10 ml) hexanoic acid (0.63 ml, 5 mmol), *N,N*′‐dicyclohexylcarbodiimide (1.03 g, 5 mmol) and *N,N*‐dimethylpyridin‐4‐amine (0.016 g, 0.1 mmol) were added. The reaction mixture was stirred for 3 h at room temperature. After the reaction was completed (TLC), the formed precipitate of *N,N*′‐dicyclohexylurea was filtered, and the filtrate was concentrated under reduced pressure. The residue was treated with acetonitrile and the mixture of mono‐, di‐ and tri‐*O*‐hexanoyluridines precipitated as a white solid. The precipitate was filtered, dried to afford 0.5 g of hexanoyluridines. HPLC‐MS analysis indicated that the mixture consisted of 7% of mono‐*O*‐hexanoyluridine, 21% of di‐*O*‐hexanoyluridine and 72% of tri‐*O*‐hexanoyluridine. UV (CH_3_OH) λ_max_ 256 nm. MS (ESI^+^): *m/z* 341.00 [M−H]^−^, 439.05 [M−H]^−^, 537.10 [M−H].

### Synthesis of R/S‐1‐phenylethyl esters (Scheme S1)

#### Synthesis of 1‐phenylethyl acetates (**3** and **4**)

Enantiomerically pure (*R*)‐1‐phenylethanol or (*S*)‐1‐phenylethanol (200 mg, 1.64 mmol) and DMAP (2 mg, 16.4 μmol) were dissolved in acetic anhydride (310 μl, 3.3 mmol) and heated for 30 min at 55°C. The reaction mixture was monitored by thin‐layer chromatography (TLC, eluent chloroform). After the reaction was completed, the formed acetic acid was evaporated under reduced pressure. The residue was purified by column chromatography (silica gel, hexane/chloroform mixture, 10:1→10:5). After removal of solvents in vacuum, the desired products were obtained as colourless oils.

##### *(R*)‐1‐phenylethyl acetate (**3**)

Yield 261 mg (97%), colourless oil, *R*_f_ = 0.68 (CHCl_3_)_._^1^H NMR (CDCl_3_): δ = 1.57 (d, 3H, *J *=* *6.6 Hz, CH_3_), 2.10 (s, 3H, CH_3_); 5.92 (q, 1H, *J *=* *6.6 Hz, CH), 7.27–7.44 (m, 5H, C_6_H_5_). ^13^C NMR (CDCl_3_): δ = 21.37, 22.22, 72.32, 126.11, 127.88, 128.51, 141.69, 170.34.

##### *(S*)‐1‐phenylethyl acetate (**4**)

Yield 219 mg (81%), colourless oil, *R*_f_ = 0.69 (CHCl_3_)_._^1^H NMR (CDCl_3_): δ = 1.57 (d, 3H, *J *=* *6.6 Hz, CH_3_), 2.10 (s, 3H, CH_3_); 5.91 (q, 1H, *J *=* *6.6 Hz, CH), 7.27–7.44 (m, 5H, C_6_H_5_). ^13^C NMR (CDCl_3_): δ  =  21.37, 22.22, 72.32, 126.10, 127.88, 128.51, 141.69, 170.35.

NMR spectra were consistent with spectra of *(R*)‐1‐phenylethyl acetate reported in literature (Tielmann *et al*., 2003).

#### Synthesis of 1‐phenylethyl benzoates (**5** and **6**)

Enantiomerically pure (*R*)‐1‐phenylethanol or (*S*)‐1‐phenylethanol (200 mg, 1.64 mmol) and triethylamine (0.23 ml, 1.64 mmol) were dissolved in dichloromethane (2 ml). After the solution was cooled to an ice bath, benzoyl chloride (0.19 ml, 1.64 mmol) was added, and the reaction mixture was stirred for 1 h at ambient temperature. After the reaction was completed (TLC), the solvent was evaporated under reduced pressure. The residue was purified by column chromatography (silica gel, hexane/chloroform mixture, 10:0→10:5). The solvents were removed in vacuum, and the desired products were obtained as colourless oils.

##### *(R*)‐1‐phenylethyl benzoate (**5**)

Yield 184 mg (50%), colourless oil, *R*_f_ = 0.75 (CHCl_3_)_._^1^H NMR (CDCl_3_): δ = 1.71 (d, 3H, *J *=* *6.6 Hz, CH_3_), 6.17 (q, 1H, *J *=* *6.6 Hz, CH), 7.31–7.52 (m, 8H, CH=CH), 8.10–8.16 (m, 2H, CH=CH). ^13^C NMR (CDCl_3_): δ = 22.45, 72.94, 126.07, 127.92, 128.36, 128.58, 129.67, 130.54, 132.95, 141.81,165.84.

##### *(S*)‐1‐phenylethyl benzoate (**6**)

Yield 360 mg (97%), colourless oil, *R*_f_ = 0.76 (CHCl_3_)_._^1^H NMR (CDCl_3_): δ = 1.71 (d, 3H, *J *=* *6.6 Hz, CH_3_), 6.17 (q, 1H, *J *=* *6.6 Hz, CH), 7.31–7.53 (m, 8H, CH=CH), 8.09–8.15 (m, 2H, CH=CH). ^13^C NMR (CDCl_3_): δ = 22.44, 72.93, 126.07, 127.92, 128.36, 128.57, 129.67, 130.55, 132.95, 141.81,165.84.

^1^H NMR spectrum was consistent with spectrum of 1‐phenylethyl benzoate racemic ester reported previously (Bellezza *et al*., 2000).

#### Synthesis of 1‐phenylethyl hexanoates (**7** and **8**)

Enantiomerically pure (*R*)‐1‐phenylethanol or (*S*)‐1‐phenylethanol (400 mg, 3.28 mmol), hexanoic acid (205 μl, 1.64 mmol), DCC (371 mg, 1.8 mmol) and DMAP (20 mg, 0.164 mmol) were dissolved in 10 ml of dichloromethane and stirred for 3 h at ambient temperature. After the reaction was completed (TLC), the formed precipitate was filtered, and the solvent was removed under reduced pressure. The residue was purified by column chromatography (silica gel, hexane/chloroform mixture, 10:0→10:5). After removal of solvents in vacuum, the desired products were obtained as colourless oils.

##### (*R*)‐1‐phenylethyl hexanoate (**7**)

Yield 288 mg (80%), colourless oil, *R*_f_ = 0.82 (CHCl_3_)_._^1^H NMR (CDCl_3_): δ = 0.91 (t, 3H, *J *=* *6.9 Hz, CH_3_), 1.25–1.42 (m, 4H, 2CH_2_), 1.56 (d, 3H, *J *=* *6.6 Hz, CH_3_), 1.61–1.70 (m, 2H, CH_2_), 2.33–2.38 (m, 2H, CH_2_), 5.93 (q, 1H, *J *=* *6.6 Hz, CH), 7.28–7.41 (m, 5H, CH=CH). ^13^C‐NMR (CDCl_3_): δ = 13.91, 22.28, 22.32, 24.67, 31.27, 34.60, 72.01, 126.07, 127.79, 128.47, 141.88, 173.11.

##### *(S*)‐1‐phenylethyl hexanoate (**8**)

Yield 272 mg (75%), colourless oil, *R*_f_ = 0.82 (CHCl_3_)_._^1^H NMR (CDCl_3_): δ = 0.91 (t, 3H, *J *=* *6.9 Hz, CH_3_), 1.27–1.40 (m, 4H, 2CH_2_), 1.56 (d, 3H, *J *=* *6.6 Hz, CH_3_), 1.61–1.70 (m, 2H, CH_2_), 2.32–2.38 (m, 2H, CH_2_), 5.93 (q, 1H, *J *=* *6.6 Hz, CH), 7.28–7.42 (m, 5H, CH=CH). ^13^C‐NMR (CDCl_3_): δ  =  13.91, 22.28, 22.32, 24.67, 31.27, 34.60, 72.01, 126.07, 127.79, 128.47, 141.88, 173.11.

NMR spectra were consistent with spectra of 1‐phenylethyl hexanoate racemic ester reported in literature (Lourenço *et al*., 2015).

### Environmental samples, DNA extraction and construction of the metagenomics libraries

Metagenomic libraries were constructed from soil and sediment samples using a pUC19 vector (Table S1). For DNA isolation directly from soil or sediment ‘ZR Soil Microbe DNA MidiPrep™’ was used. The total DNA was partially digested with restriction endonuclease selected from the list: PstI, HindIII or BamHI. The different restriction endonuclease were used to obtain a larger diversity of the DNA fragments which were inserted into the pUC19 vector and used to transform *E. coli* DH5alpha competent cells by electroporation as described (Stanislauskienė *et al*., 2018). To analyse the number of clones in the library, quality of the library (a ratio of white/blue colonies) and the average insert length, an aliquot of the transformed cells was diluted and spread on LB agar (10 g l^−1^ tryptone, 5 g l^−1^ yeast extract, 10 g l^−1^ NaCl and 15 g l^−1^ agar) supplemented with the appropriate antibiotics (40 μg ml^−1^ kanamycin, 100 μg ml^−1^ ampicillin), 1 mM IPTG and 1 mM X‐gal. Ten to twenty of individual white colonies‐forming clones were randomly chosen for plasmid DNA isolation by Thermo Scientific™ GeneJET Plasmid Miniprep Kit (Thermo Fisher Scientific) and analysis of the length of the insert. The remaining undiluted mixture of the cells was spread on LB agar (10 μl of the bacterial suspension per one Petri dish (92 mm)) supplemented with 40 μg ml^−1^ kanamycin and 100 μg ml^−1^ ampicillin. After cultivation for 14–16 h, a biomass was scraped from agar, and total plasmid DNA was isolated by Thermo Scientific™ GeneJET Plasmid Midiprep Kit (Thermo Fisher Scientific). The obtained DNA mixture (a metagenomics library) was stored at –20°C and used for a further transformation of the *E*. *coli* DH10B Δ*pyrFEC::Km* cells.

### Functional screening of the metagenomics libraries

#### Screening of esterases by using acylated uridine derivatives on agar plates

The clones exhibiting acetylesterase activity were identified on a MD medium (33.9 g l^−1^ Na_2_HPO_4_ 15 g l^−1^ KH_2_PO_4_, 5 g l^−1^ NH_4_Cl, 2.5 g l^−1^ NaCl, 2% (w/v) glucose, 0.2% Casamino acid, 1 mM IPTG) containing 100 μg ml^−1^ ampicillin and 40 μg ml^−1^ kanamycin, 0.02 mg ml^−1^ 2′,3′,5′‐*O*‐acetyluridine (**1**) or 2′,3′,5′‐*O*‐hexanoyluridine (**2**) as the sole source of uridine, allowing only the growth of recombinants that can complement the uridine auxotrophy of the *E. coli* DH10B Δ*pyrFEC*::Km strain by hydrolysing the uridine esters.

#### Screening of esterases by using the tributyrin‐supplemented agar plates

LB agar medium supplemented with 1% tributyrin was used to screen for lipolytic/esterolytic activity. The clones showing a halo around the individual colonies, which indicated hydrolysis of the tributyrin, were selected on the emulsified tributyrin medium after 1–2 days of growth at 37°C. (Popovic *et al*., 2017) Peña‐García *et al*., 2016; Ranjan *et al*., 2005)

### DNA sequencing and gene annotation

Nucleotide sequences were determined at Macrogen Europe (Netherlands) and using the following sequencing primers: M13F‐pUC (5′‐GTTTTCCCAGTCACGAC‐3′), M13R‐pUC (5′‐CAGGAAACAGCTATGAC‐3′), T7 Promoter (5′‐ TAATACGACTCACTATAGGG‐3′), T7 terminator (5′‐TAATACGACTCACTATAGGG‐3′) or LIC Reverse Sequencing primer, 24‐mer (5′‐GAGCGGATAACAATTTCACACAGG‐3′). Some individual clones contained more than one ORF in each DNA fragment. ORFs were analysed by using the Unipro UGENE program, and homology search was conducted using the Blast server (http://www.ncbi.nlm.nih.gov/BLAST). For further analysis the ORFs encoding putative hydrolases were chosen. When homology search did not predict a hydrolase, the deletion analysis to obtain the truncated variants of the plasmid and the functional reselection on the appropriate substrate was carried out. To confirm that the hits encoded the enzymes with an esterolytic activity, the selected genes were PCR‐amplified, and the resulting fragments were ligated into pET21a or pLATE31 expression vectors. Phylogenetic analysis was conducted using the *Maximum Likelihood Tree* routine of mega 7 software.(Kumar *et al*., 2016; Thompson *et al*., 1994) The alignment was performed using clustalw in mega 7.

### Expression vectors and PCR primers

The genes of the selected enzymes were amplified with Phusion DNA polymerase using primers (Table S2). Metagenomic esterase‐encoding genes of 12T, 24T5, 45T3, 33T1, and 3T clones were ligated into pET21a, and all other genes were ligated into pLATE31. *E. coli* cells transformed with recombinant plasmids were cultivated on LB agar supplemented with 100 μg ml ampicillin.

### Overexpression and purification of esterases

The recombinant proteins were overexpressed in *E. coli* strain BL21 (DE3). *E. coli* cells were grown in 20 ml BHI (Brain‐Heart‐Infusion Broth) medium containing ampicillin (100 mg ml) at 37°C with aeration. Protein expression was induced by adding 0.5 μM IPTG at 0.6–1 OD_600_, and cells were grown for a further 4–18 h at 30°C. Cells expressing 12T and 3T were grown at 23°C after induction to increase protein solubility. Wet cell biomass from 20 ml culture broth were suspended in 5–10 ml of buffer A (50 mM potassium phosphate, pH 7.5) and disrupted by sonication for 2.5 min. A lysate was cleared by centrifugation at 15 000 × *g* for 4 min. Cleared lysate was applied to 0.2 ml HisPur™ Ni‐NTA spin column (equilibrated with the buffer A). The column was washed with the buffer A, and the proteins were eluted with buffer A containing 300 mM imidazole. The active fractions were combined and dialyzed against the buffer B (50 mM potassium phosphate, pH 7.5), at 25°C. All the purification procedures were performed at room temperature.

### Proteins concentration and purity determination

The concentration of protein was determined using Pierce™ Coomassie Plus (Bradford) Assay Reagent by Standard Microplate Protocol. Proteins were analysed by sodium dodecyl sulfate polyacrylamide gel electrophoresis (SDS–PAGE, 14% separating and 4.0% stacking) according to Laemmli. Gels were developed in Coomassie Brilliant Blue G‐250 dye, scanned in 16 bit format and quantified by GelAnalyser program. Each sample contained 2 μg of total protein. Quantities of impurities and target proteins were estimated using calibration curve generated from known amounts of BSA: 0.125, 0.25 and 0.5 μg per band. The purity of analysed protein was calculated as the ratio between quantity of target protein and quantity of all proteins.

### Enzymatic activity of esterases

#### Hydrolysis of pNp esters

The activity of esterase were assayed by incubating the enzyme with 1 mM pNP‐substrate (from 10 mM stock in DMSO) in 50 mM potassium phosphate, pH 7.5, buffer at a 37°C for 10 min in 100 μl reaction volume. 5–300 ng of protein, depending on the enzyme specificity for the substrate, were present into the reaction mixture. The absorbtion of the reaction mixture at 405 nm was measured against enzyme‐free blank to compensate for the substrate auto‐hydrolysis (Hernández‐García *et al*., 2017; Beisson *et al*., 2000). One unit is defined as the amount of enzyme that catalyses the formation of 1 μmol of 4‐nitrophenol (molar extinction coefficient *ε* = 12.3 M^−1 ^cm^−1^) per minute. Enzyme activity was tested against different pNP‐acyl esters [acetate, butyrate, valerate, decanoate, palmitate and stearate].

#### Hydrolysis of nitrocefin

Nitrocefin is a chromogenic cephalosporin substrate routinely used to detect the presence of beta‐lactamase enzymes (Petersen *et al*., 2001; Ohlhoff *et al*., 2015; Chow *et al*., 2013). Once hydrolyzed, the degraded nitrocefin compound rapidly changes colour from yellow to red. A hydrolytic activity was assayed in 50 mM potassium phosphate buffer, pH 7.0, containimg 1 mM nitrocefin at 37°C for 2 h. Total reaction volume was 50 μl. The change of the colour was evaluated visually.

#### pH‐indicator‐based assay

A hydrolytic activity was assayed in reaction mixture containing 5 mM potassium phosphate buffer, pH 7.5, 0.5 mM Phenol Red (from 10 mM stock in water), 1 μl enzyme (0.1–4.6 μg/reaction) and 10 mM substrate: *R/S*‐1‐phenylethyl acetate/hexanoate/benzoate (from 100 mM stock in acetone), pentaacetylglucose or pentaacetylgalactose (from 100 mM stock in acetone), 3′‐*O*‐acetyl‐2′‐deoxyuridine, 3′‐*O*‐acetyl‐*N* ^4^‐benzoyl‐2′‐deoxycytidine, 3′‐*O*‐levulinyl‐N^4^‐benzoyl‐2′‐deocytidine, and 5′‐*O*‐levulinyl‐*N* ^4^‐benzoyl‐2′‐deocytidine (100 mM stock in DMSO). The total reaction mix volume was 100 μl. Reaction mixture was incubated at room temperature up to 5 h. A change of colour from red to yellow indicated the hydrolysis of esters (Martínez‐Martínez *et al*., 2018; Janes *et al*., 1998; Lee *et al*., 2016).

#### Hydrolysis products test by thin‐layer chromatography (TLC) method

A hydrolytic activity was assayed in reaction mixture containing 45 mM potassium phosphate buffer, pH 7.5, 1 μl enzyme (0.1–4.6 μg/reaction) and 10 mM substrate: β‐d‐glucose pentaacetate*,* β‐d‐ galactose pentaacetate (from 100 mM stock in acetone), 3′‐*O*‐benzoyl‐2′deoxyuridine (10 mg ml^−1^ stock in DMF), 3′‐*O*‐levulinyl‐*N* ^4^‐benzoyl‐2′deocytidine and 5′‐*O*‐levulinyl‐*N* ^4^‐benzoyl‐2′‐deocytidine (from 100 mM stock in DMSO). The total reaction mix volume was 20 μl. Reaction mixture was incubated at 30°C temperature up to 3 h. Thin‐layer chromatography (TLC) was conducted on the Merck silica gel 60F254 plates, using the dichloromethane and methanol (9:1) mixture of solvents. β‐d‐glucose pentaacetate *and* β‐d‐ galactose pentaacetate were visualized by anisaldehide stain (50 ml ethanol, 1.9 ml of concentrated sulfuric acid, 0.54 ml of glacial acetic acid and 0.14 ml of *p*‐anisaldehyde). The plate was developed by heating on a hot plate. Synthetic nucleosides were exposing to UV light.

## Conflicts of interest

There are no conflicts to declare.

## Supporting information


**Table S1.** Metagenomic libraries used in this work.
**Table S2.** Primers used for amplification of genes of selected hydrolases.
**Table S3.** Predicted signal peptide sequences identified in the selected esterases by bioinformatics.
**Fig. S1.** Selected clones on MD medium, MD+ compound **1,** MD+compound **2** and MD+uridine after 2 days of incubation at 37°C.
**Fig. S2.** The alignment was performed by ClustalW software.
**Fig. S3.** Analysis of the purified esterases by SDS‐PAGE.
**Fig. S4**. LB medium with tributyrin plates after 2 days of incubation at 37°C.
**Scheme S1.** Synthesis of optically active esters (**3**–**8**).
