# Supplementary material for: Application of the uridine auxotrophic host and synthetic nucleosides for a rapid selection of hydrolases from metagenomic libraries
Source: Microb Biotechnol. 2018 Oct 9;12(1):148–60. doi: 10.1111/1751-7915.13316 (PMC6302743; doi:10.1111/1751-7915.13316)
Supplement: Supplementary file 1 — Table S1. Metagenomic libraries used in this work. Table S2. Primers used for amplification of genes of selected hydrolases. Table S3. Predicted signal peptide sequences identified in the selected esterases by bioinformatics. Fig. S1. Selected clones on MD medium, MD+ compound 1, MD+compound 2 and MD+uridine after 2 days of incubation at 37°C. Fig. S2. The alignment was performed by ClustalW software. Fig. S3. Analysis of the purified esterases by SDS‐PAGE. Fig. S4. LB medium with tributyrin plates after 2 days of incubation at 37°C. Scheme S1. Synthesis of optically active esters (3–8). [file MBT2-12-148-s001.docx]

**Supporting Information**

**Application of the uridine auxotrophic host and synthetic nucleosides for a rapid selection of hydrolases from metagenomic libraries**

Nina Urbelienė, Simonas Kutanovas, Rita Meškienė, Renata Gasparavičiūtė, Daiva Tauraitė, Martyna Koplūnaitė, Rolandas Meškys

*Department of Molecular Microbiology and Biotechnology, Institute of Biochemistry, Life Sciences Center, Vilnius University, Sauletekio 7, Vilnius LT-10257, Lithuania.*

For correspondence: E-mail [nina.urbeliene@bchi.vu.lt](mailto:nina.urbeliene@bchi.vu.lt), E-mail [rolandas.meskys@bchi.vu.lt](mailto:rolandas.meskys@bchi.vu.lt)

# Supplementary Tables

**Table S1** Metagenomic libraries used in this work. The clones selected using compound **2** as the uridine source are marked by asterisk.

| **Metagenome library** | **DNA source** | **Vector/insertion site** | **Approx. number of clones in library** | **Number of selected hits (names of hits)** |
| --- | --- | --- | --- | --- |
| L500 | Soil enriched in the presence of pentaacetylglucose | pUC19/PstI | 1,200,000 | 3 (24T5, 24T1, 24T3) |
| 5CRK9 | Soil | pUC19/PstI | 170,000 | 2 (33T1, 33T3) |
| KAU | Soil | pUC19/PstI | 220,000 | 3 (30T1, 30T2, 45T3) |
| RK7 | Soil | pUC19/PstI | 1,120,000 | 3 (36T1, 36T2, 3T) |
| GRU1 | Soil | pUC19/PstI | 1,770,000 | 1 (GRU1) |
| 442 | Soil | pUC19/PstI | 472,500 | 1 (12T) |
| MO10 | Soil, pre-enrichment on mixture of ketones (gas phase) | pUC19/HindIII | 246,000 | 2 (MO101T, MO4B) |
| SVGPA | Soil | pUC19/HindIII | 100,000 | 3 (SVG1 SVG3, SVGPA2T) |
| K3* | Soil enriched in the presence of amines | pUC19/BamHI | 20,000 | 1 (K3H2) |
| BD2* | Soil | pUC19/PstI | 20,000 | 1 (BD2H) |
| BD | Soil | pUC19/PstI | 30,000 | 2 (BD1, BD9) |
| PLA1 | Sediments | pUC19/BamHI | 400,000 | 1 (PLA1) |
| EN1* | Soil enriched in the presence of aromatic amines | pUC19/HindIII | 20,000 | 1 (EN1H) |
| EN2* | Soil enriched in the presence of aromatic amines | pUC19/HindIII | 20,000 | 1 (EN3H) |
| CAP1* | Soil | pUC19/PstI | 80,000 | 1 (CAP3H) |
| 1315* | Soil | pUC19/HindIII | 20,000 | 1 (1315H) |
| RIE | Soil enriched in the presence of aromatic amines | pUC19/HindIII | 10,000 | 1 (RIEB) |
| 4H | Soil enriched in the presence of aromatic amines | pUC19/HindIII | 10,000 | 1 (4H1T) |
| CBD | Soil | pUC19/PstI | 24,000 | 1 (C233) |

Soil samples were from Vilnius region, Lithuania (54°40’N25°16’E); sediments were from Lake Plateliai, Plunge district, Lithuania (56° 2'N21° 51')

**Table S2** Primers used for amplification of genes of selected hydrolases. The 5'-end of the primer highlighted Italic underline font includes pLATE31 vector-specific sequences for the ligation independent cloning.

| Clone | F primer | R primer |
| --- | --- | --- |
| 24T5 | TACATATGCTGAGAAAATGGCTG | CTAAGCTTGTGCGCTTCGATGAAG |
| 24T1 | *AGAAGGAGATATAACTATGG*ATCAAACACTTCTCGCTT | *GTGGTGGTGATGGTGATGGCC*CTCTGCGAGGTAAGGCTT |
| 24T3 | *AGAAGGAGATATAACTATGG*AAAGCCAGACGTTTGGGA | *GTGGTGGTGATGGTGATGGCC*CTTCAGGCTCTCCGCAA |
| 33T1 | TACATATGAAAGCCAGACGTTTG | TACTCGAGTCTTCAGGCTCTCAGCAAAG |
| 30T1 | *AGAAGGAGATATAACTATG*CGTCGTCCATCCTTG | *GTGGTGGTGATGGTGATGGCC*CTGGGCCAGATGCTCG |
| 30T2 | *AGAAGGAGATATAACTATG*AAAGTTAAAATTTTAATAGTTTTCC | *GTGGTGGTGATGGTGATGGCC*TTGAGTAATTTTATTTTC |
| 36T2 | *AGAAGGAGATATAACTATG*ACCCTGAGACTTGCG | *GTGGTGGTGATGGTGATGGCC*CAGGTTGTCGCGGAAC |
| 3T | ATGCATATGGCGTTTTTCGATTTGCC | ATGCTCGAGATCCAGCAGATCATGCAG |
| GRU1 | *AGAAGGAGATATAACTATG*CCGGTTATCGACATG | *GTGGTGGTGATGGTGATGGCC*CAAACCAAGCATAAACTGAAC |
| BD1 | *AGAAGGAGATATAACTATG*ATCAGAAACATCTTTGGC | *GTGGTGGTGATGGTGATGGCC*CTGCAGACCACGGCG |
| 12T | TACATATGCTTCATCGTCATTGC | TAGTCGACGAGTTTGGTCGCGGGATC |
| 33T3 | *AGAAGGAGATATAACTATG*AATAAATCAAAACGTACGT | *GTGGTGGTGATGGTGATGGCC*TTTGAAAATGGACAGATCTATC |
| BD9 | *AGAAGGAGATATAACTATG*AAGCCCACTCGCTG | *GTGGTGGTGATGGTGATGGCC*GAACAGCGCAGCGTC |
| 36T1 | *AGAAGGAGATATAACTATG*ATAAAACTCAATCCTGTCTTC | *GTGGTGGTGATGGTGATGGCC*GAAGAACAGCCGCGG |
| 45T3 | GGCGAGTTGCATATGACCC | GGAAGCTTGCCCGTGGGAAGG |
| C233 | *AGAAGGAGATATAACTATG*ACGAACCCCAAGTCG | *GTGGTGGTGATGGTGATGGCC*GACCTCCGTGCTGTCG |
| PLA1 | *AGAAGGAGATATAACTATG*CGCCGCCGC | *GTGGTGGTGATGGTGATGGCC*CGGCTTTGCCATCACC |
| MO101T | *AGAAGGAGATATAACTATG*AACATCGTATTGATTCA | *GTGGTGGTGATGGTGATGGCC*TAAAGGCACTGAAATATTTCG |
| SVG1 | *AGAAGGAGATATAACTATG*AGTCTCCACCTCAAGTG | *GTGGTGGTGATGGTGATGGCC*CGGTTTGGCGTAGACC |
| SVG3 | *AGAAGGAGATATAACTATG*CGCAAGGGG | *GTGGTGGTGATGGTGATGGCC*GTTAAAGACAGAATCAAGAAAC |
| B11 | *AGAAGGAGATATAACTATG*CGCGATGCCTC | *GTGGTGGTGATGGTGATGGCC*GTGCGCTTCGATGAAG |
| 4H1T | *AGAAGGAGATATAACTATG*AGAATCATTTTATCCCT | *GTGGTGGTGATGGTGATGGCC*TACTTTAGAAAAGAAGTCCTGA |
| RIEB | *AGAAGGAGATATAACTATG*CGCACATCTTTGC | *GTGGTGGTGATGGTGATGGCC*GAGGTGCGCCGTCA |
| MO4B | *AGAAGGAGATATAACTATG*AAAAGTCCATCCAAG | *GTGGTGGTGATGGTGATGGCC*ACGTAAGGCCGTACTG |
| EN1H | *AGAAGGAGATATAACTATG*CAATCAACCAA | *GTGGTGGTGATGGTGATGGCC*CGAATAGATTTTTTCAGATA |
| EN3H | *AGAAGGAGATATAACTATG*CCCAACAACATCG | *GTGGTGGTGATGGTGATGGCC*GGACACATCTTCCCCC |
| BD2H | *AGAAGGAGATATAACTATG*AGTTCGCTCCGCC | *GTGGTGGTGATGGTGATGGCC*AAGCGTCCTCTCAAACCA |
| CAP3H | *AGAAGGAGATATAACTATG*CATCGAGCTGTTTC | *GTGGTGGTGATGGTGATGGCC*CGGCGTACACGGC |
| 1315H | *AGAAGGAGATATAACTATG*TGGGCCACCAGC | *GTGGTGGTGATGGTGATGGCC*CGAGACGGACTTGCCTAA |
| SVGPA-2T | *AGAAGGAGATATAACTATG*CCGGCGCTTGACGGC | *GTGGTGGTGATGGTGATGGCC*CGGCCGTCATCCG |
| K3H2 | *AGAAGGAGATATAACTATG*TCCGACCCGCAA | *GTGGTGGTGATGGTGATGGCC*GCCGGCGAGCGCTTCCTT |
| Tb_10-7T | *AGAAGGAGATATAACTATG*AATCATAACGTATCCGC | *GTGGTGGTGATGGTGATGGCC*ACGGTCGAGAAATTTCAAG |
| Tb_7_1T | *AGAAGGAGATATAACTATG*ACACATGGATTCGACA | *GTGGTGGTGATGGTGATGGCC*GTCATCAATCGCGGC |

**Table S3 Predicted** signal peptide sequences identified in the selected esterases by bioinformatics (<http://smart.embl.de>; <http://www.csbio.sjtu.edu.cn/bioinf/Signal-3L/>). The differences in sequences predicted by two methods are underlined.

| Clone | Signal peptide range | | Predicted signal peptide sequence | Detection in extracellular space |
| --- | --- | --- | --- | --- |
|  | **SMART** | **Signal-3L 2.0** |  |  |
| 24T5 | 1-21 | 1-21 | MLRKWLLASVMLAAIVAPAAA | No |
| 33T1 | 1-26 | 1-26 | MKARRLGSSLIMAGIAIFAASVAVRA | Yes |
| 12T | 1-28 | 1-28 | MLHRHCRPRHAAWLAIGLLTVVSLNLRA | No |
| 24T3 | 1-26 | 1-26 | MKARRLGSVLLVAGVAIFAASVAAKA | Yes |
| 24T1 | 1-23 | 1-21 | MIKHFSLALASAVLLTGAAFADA | Yes |
| 3T | - | - | not predicted | n.a. |
| MO101T | - | - | not predicted | No |
| SVG3 | 1-25 | 1-25 | MRKGQVAVFYAVFFIVSLFSQQVFA | Yes |
| CAP3H | 1-23 | 1-17 | MHRAVSQAALFVVAGLLLAGCGG | No |
| 4H1T | 1-19 | 1-19 | MRIILSLLAIFLSSGLVQA | No |
| EN3H | - | - | not predicted | No |
| 45T3 | - | - | not predicted | n.a. |
| 30T1 | 1-20 | 1-20 | not predicted | No |
| 1315H | - | 1-18 | MWATSFSSILRVAISISA | No |
| GRU1 | - | - | not predicted | n.a. |
| PLA1 | 1-27 | 1-26 | MRRRTLLRAAVGLLTAWGLAGIPASAG | n.a. |
| 30T2 | 1-20 | 1-20 | MKVKILIVFLMLISIFSAQA | n.a. |
| 36T1 | - | - | not predicted | n.a. |
| BD9 | 1-22 | 1-18 | MKPTRWLALLCLALPLLASCAS | n.a. |
| SVG1 | 1-21 | 1-21 | MSLHLKCAALVSALLAFPAFA | Yes |
| SVGPA2T | - | - | not predicted | No |
| K3H2 | - | - | not predicted | No |
| 33T3 | 1-23 | 1-23 | MNKSKRTFLKLLSSVAVIVITLA | No |
| RIEB | 1-22 | 1-22 | MRTSLLSATASLLLLSSGTAMA | No |
| MO4B | - | - | not predicted | No |
| 36T2 | 1-21 | 1-20 | MTLRLAALGLAALSATMAVAG | n.a. |
| EN1H | - | - | not predicted | No |
| BD2H | 1-21 | - | MSSLRRLGLLLFPLFTVPLHA | No |
| C233 | - | - | not predicted | No |
| BD1 | 1-29 | 1-31 | MIRNIFGTLPKSAVLLSALALSTVMTVNSQA | n.a. |
| Tb_10_7T | - | - | not predicted | Yes |
| Tb_7_1T | - | - | not predicted | No |

n.a. – not analyzed

# Supplementary Figures

**Figure S1.** Selected clones on MD medium, MD+ compound **1,** MD+compound **2** and MD+uridine after 2 days of incubation at 37°C.

**
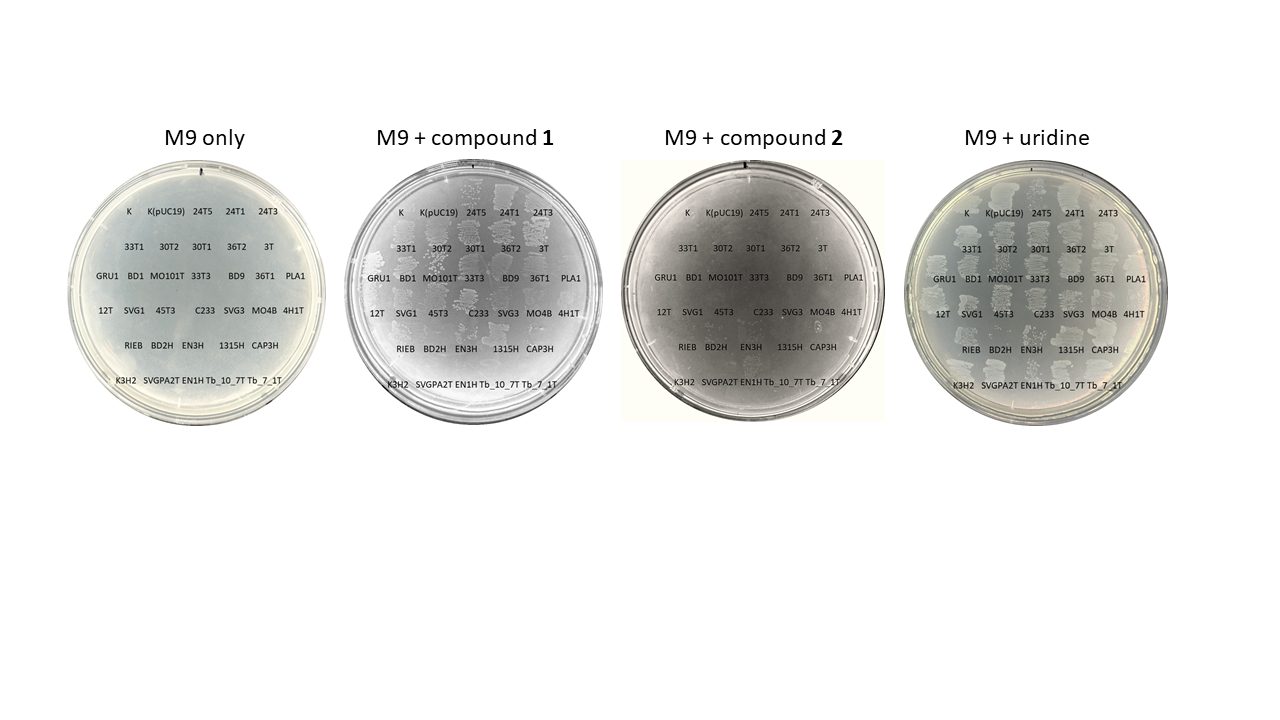
**

**Figure S2.** The alignment was performed by ClustalW software. Underlined sequences indicate conserved motifs and putative catalytic residues. **(A**) 19 selected esterses of α/β hydrolases superfamily; (**B**), EN1H and MO4B α/β hydrolases/galactose-binding domain-like esterases; (**C**), BD9, 33T3, PLA1, 36T1 SGNH hydrolases, (**D**), β-lactamases (12T and SVG1).
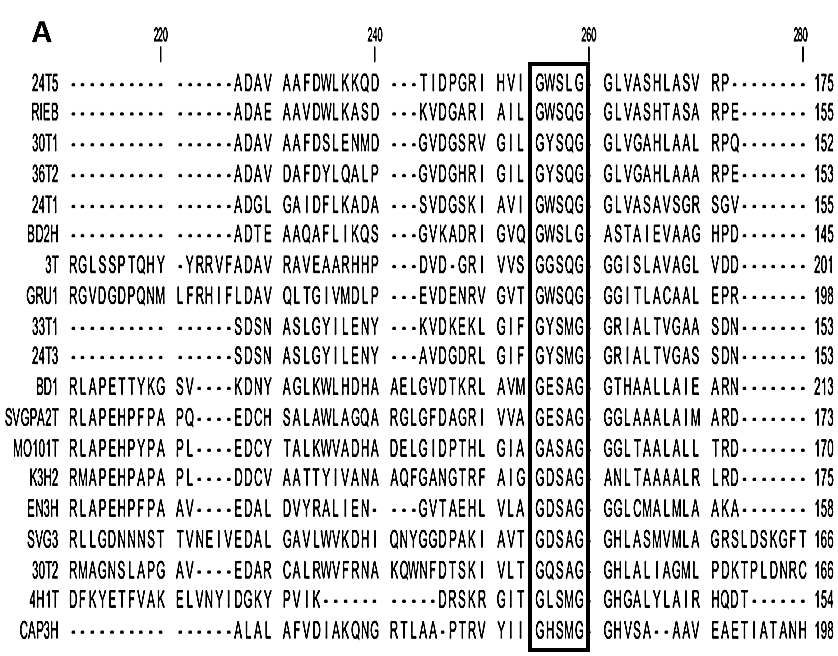


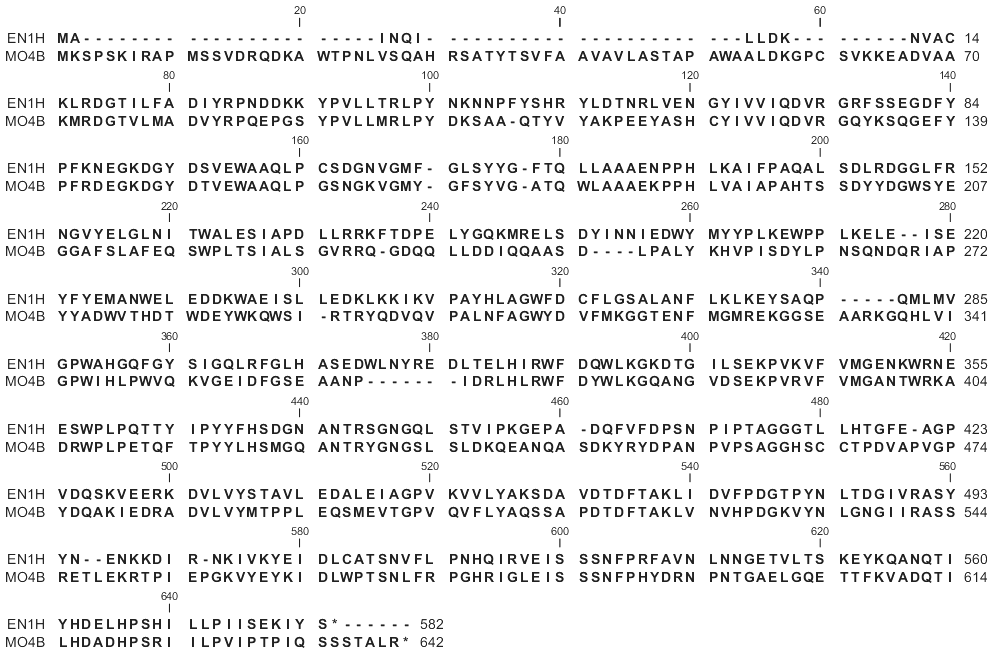


**B**


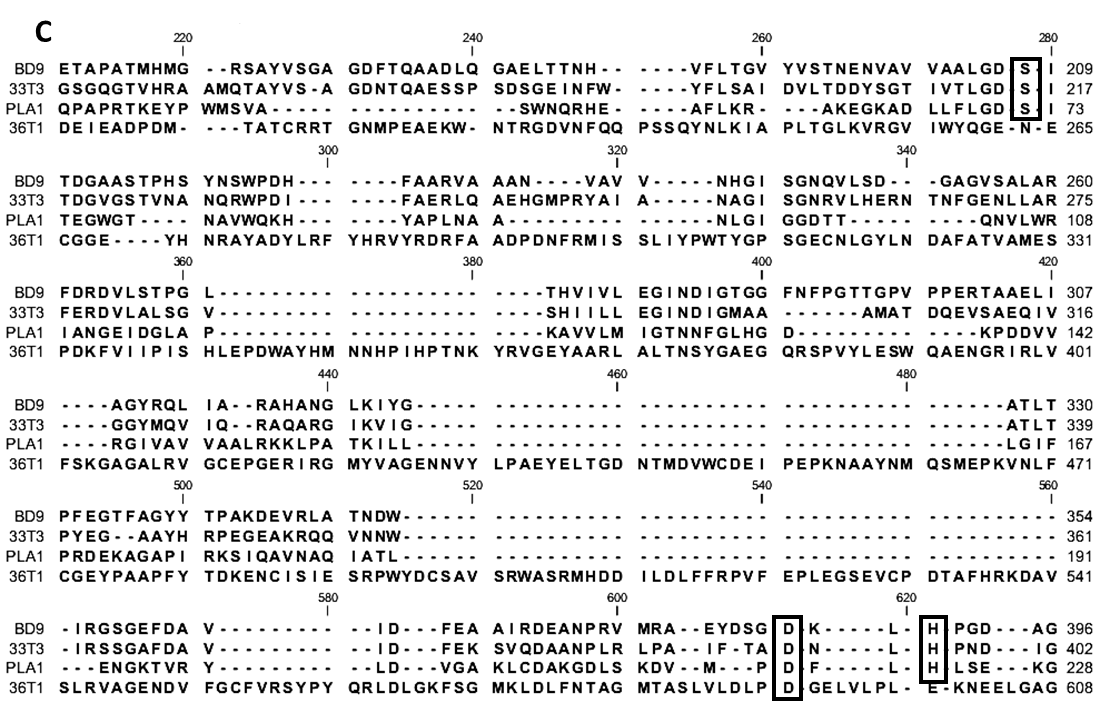

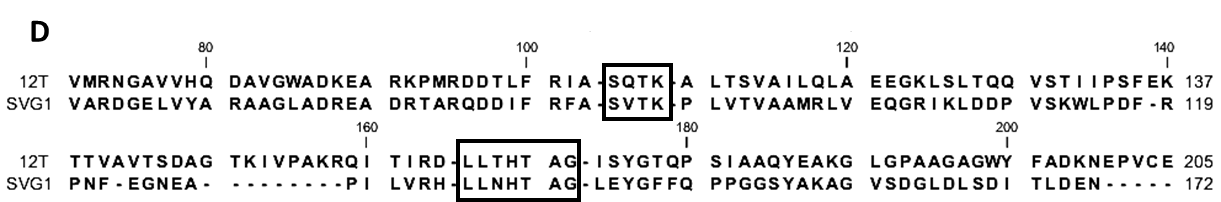


**Figure S3.** Analysis of the purified esterases by SDS-PAGE. Proteins were separate by SDS-PAGE (14% separating and 4.0% stacking) according to Laemmli. Gels were developed in Coomassie Brilliant Blue G-250 dye, scanned in 16 bit format and quantified by GelAnalyser program. Each sample contained 2 µg of total protein. Quantities of impurities and target proteins were estimated using calibration curve generated from known amounts of BSA: 0.125, 0.25 and 0.5 µg per band. Purity of analysed protein was calculated as the ratio between quantity of target protein and quantity of all proteins.


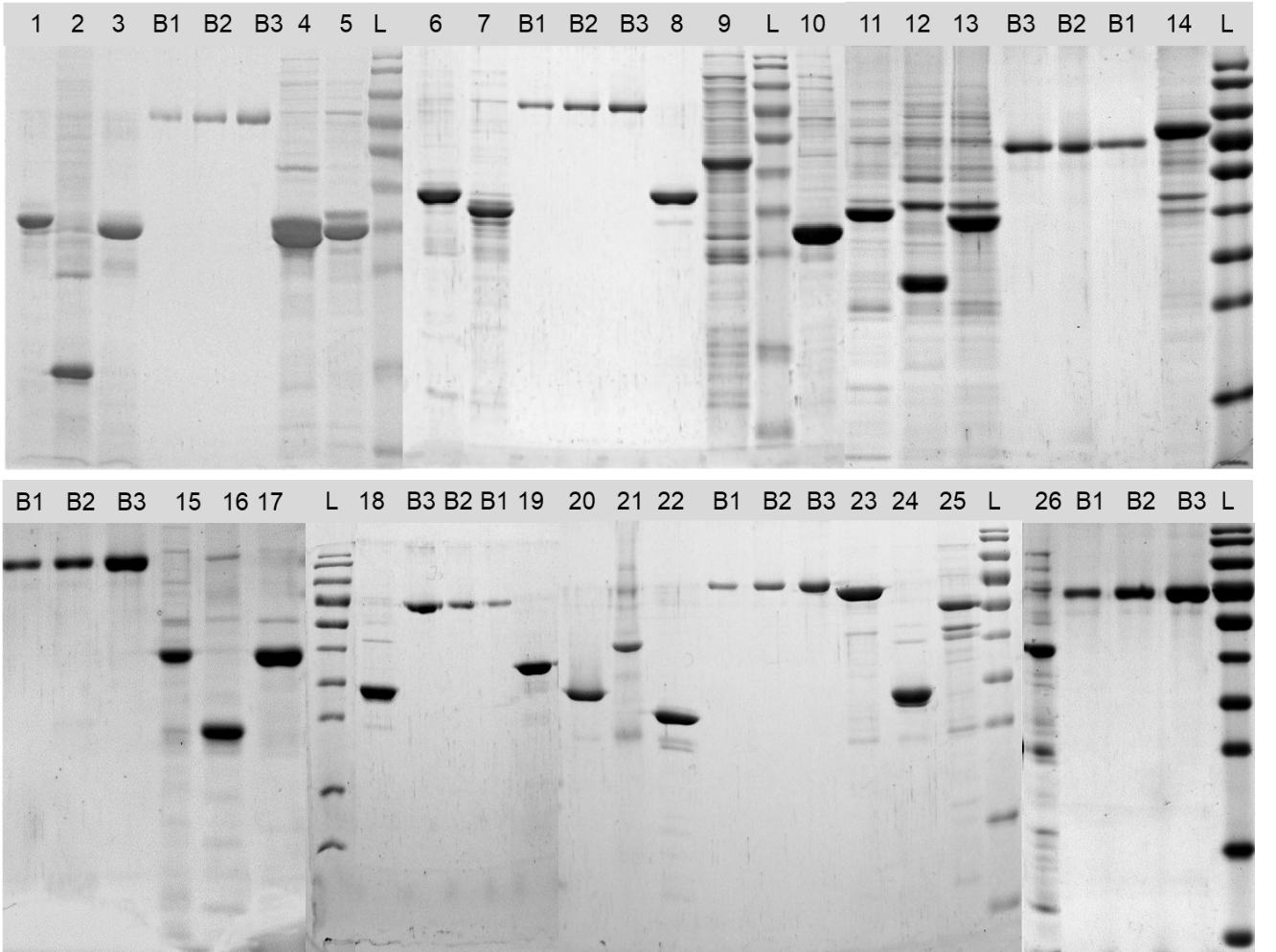


Note:

B1 – 125 ng BSA, B2 – 250 ng BSA, B3 – 500 ng BSA;

L – PageRuler^TM^ Prestained Protein Ladder.

1 – 24T5, 2- 45T3, 3 – 24T1, 4 – 24T3, 5 – 30T1; 6 – 3T, 7 – 33T1, 8 – GRU1, 9 – BD9, 10 – 30T2, 11 – SVG1, 12 – 36T2, 13 – BD1, 14 – 36T1, 15 – C233, 16 – PLA1, 17 – MO101T, 18 – EN3H, 19 – SVG3, 20 – K3H2, 21 – 33T3, 22 – BD2H, 23 – EN1H, 24 – Tb10-7, 25 – CAP3H; 26 – 12T.


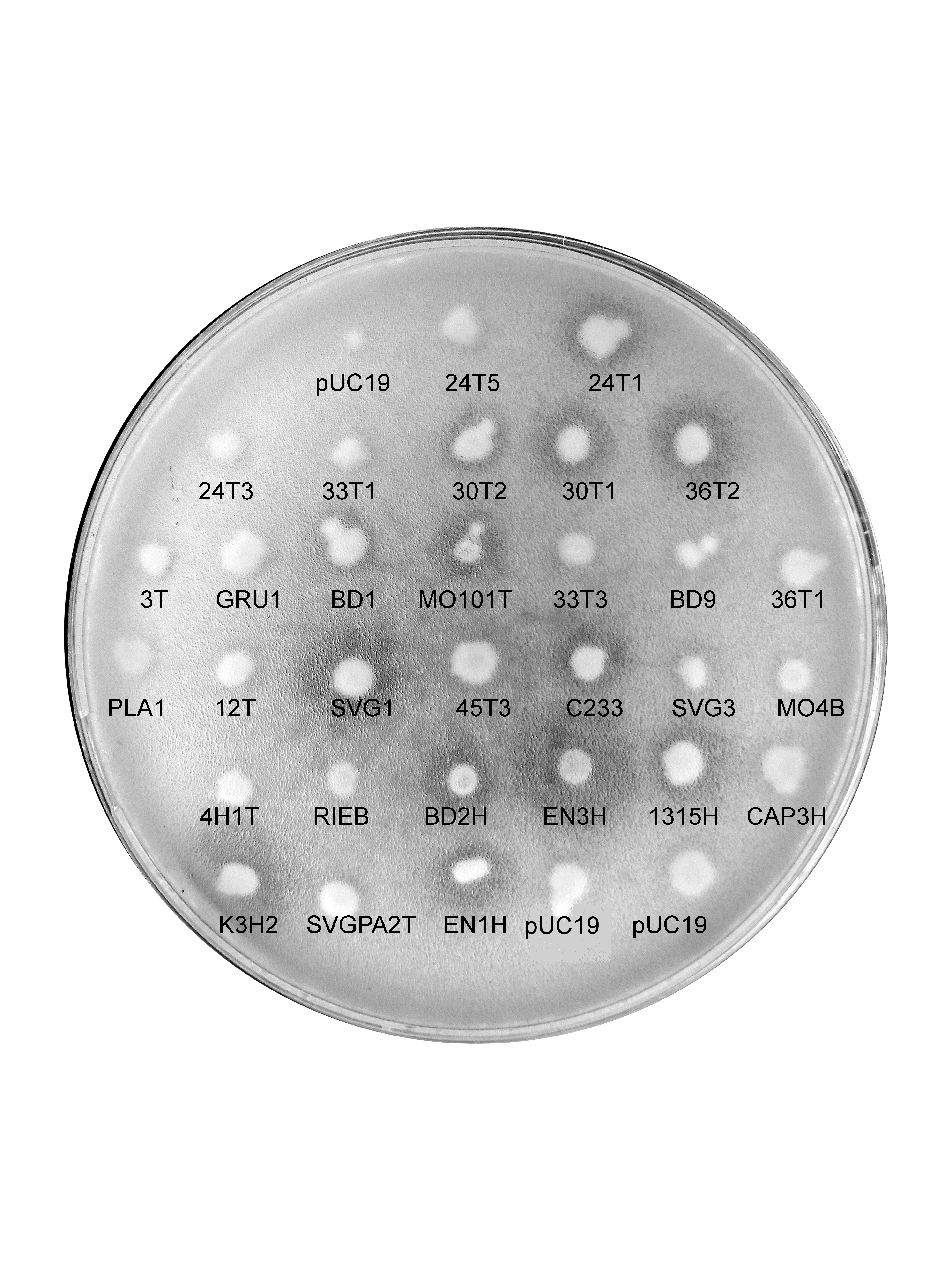
**Figure S4**. LB medium with tributyrin plates after 2 days of incubation at 37°C. A halo around individual colonies indicated hydrolysis of the tributyrin.

## Scheme of of R/S-1-phenylethyl esters synthesis

**Scheme S1.** Synthesis of optically active esters (**3**–**8**). Reagents and conditions: i) acetic anhydride, DMAP, 55 °C, 30 min; ii) benzoyl chloride, TEA, CH_2_Cl_2_, 0 °C→rt, 1 h; iii) hexanoic acid, DCC, DMAP, CH_2_Cl_2_, rt, 3 h.
